# Supplementary material for: Structural Insights into the Binding of Red Fluorescent Protein mCherry-Specific Nanobodies
Source: Int J Mol Sci. 2023 Apr 9;24(8):6952. doi: 10.3390/ijms24086952 (PMC10138814; doi:10.3390/ijms24086952)
Supplement: Supplementary file 1 [file ijms-24-06952-s001.zip › ijms-2303553-SI.pdf]

Structural insights into the binding of red fluorescent protein mCherry-specific nanobodies

Hui Liang[1], Zhiqiang Ma[1], Ziying Wang[1], Peiyu Zhong[1], Ran Li[1], He Jiang[1], Xin Zong[1], Chao Zhong[1], Xihuan Liu[1], Peng Liu[1], Jiayuan Liu[1], Haoran Zhu[1], Rui Liu[1,#], Yu Ding[1,#]

1. State Key Laboratory of Genetic Engineering, School of Life Sciences, Fudan University, Shanghai 200438, China

[#] Corresponding author: Yu Ding or Rui Liu.

Tel: +86-21-31246545

Fax: +86-21-31246544

E-mail address: yuding@fudan.edu.cn or liur@fudan.edu.cn

Materials and Reagents

Constant temperature shaking incubator (Shanghai Zhichu In.Co., China), centrifuges (Eppendorf, Germany; Thermo Fisher Scientific, US; DLAB, Beijing, China), nanodrop (Thermo Fisher Scientific, China), digital pH meter (Tianda, Shanghai, China), magnetic stirrer (DLAB, Beijing, China). AKTA GO protein purification system (Cytiva, US) and AKTA PURE protein purification system(Cytiva, US). Gryphon (Art Robbins, US). Stereo microscope (Olympus, Japan). Metal heater (DLAB, Beijing, China). Electronic balances (Tianda, Shanghai, China). Ultra-pure ddH_2O for crystal was prepared by Milli-Q (Merck Millipore, Germany). RF-20Axs fluorescence detector for HPLC (Shimadzu, Japan). ITC 200 (Malvern Panalytical, Germany), Octet Red 96 (ForteBio, US).

Amicon Ultra 10KD filter and Amicon Ultra 3KD filter (Merck Millipore, Germany),

Amicon Ultra 0.5 mL Centrifugal Filters (Merck Millipore, Germany). Slide A-lyzer mini dialysis devices (Thermo Fisher Scientific, US). Dialysis membranes MWCO-3500 (Solarbio, Beijing, China; Viskase, Germany). Superdex 200 Increase 10/300 GL, Superdex 200 Increase 5/150 GL (Cytiva, US), 5 mL prepacked desalting column (Cytiva, US). Pipette gun and tips (DLAB, Beijing, China; Eppendorf, Germany). 96-well sitting plate (Art Robbins, US). 0.45μm/0.22μm Nylon membrane (Merck Millipore, Germany). ITC syringe (Malvern Panalytical, Germany). 96 well black plate (Molecular Devices, US).

Ampicillin, kanamycin, NaCl, imidazole, isopropyl β-D-thiogalactopyranoside (IPTG), Tris-hydroxymethyl-aminomethane (TRIS), 4-(2-hydroxyethyl)piperazine-1-ethanesulfonic acid (HEPES), bromophenol blue, Coomassie brilliant blue R-250, ethylene diamine tetraacetic acid (EDTA), Nickel(II) sulfate hexahydrate from Sangon, Shanghai, China.

Agar, HCl, NaOH, ethanol, glacial acetic acid, and glycerol from Sinopharm, Shanghai, China.

Tween-20, Triton X-100, sodium dodecyl sulfate (SDS) from Solarbio, Beijing, China.

2-mercaptoethanol (2-ME), Tris-(2-carboxyethyl)-phosphine hydrochloride (TCEP) from Aladdin, Shanghai, China.

BL21(DE3) *E coli.* strains from KT, Shenzhen, China, and Abclonal, Wuhan, China.

Electrophoresis power is from Bio-Rad, US.

SDS-PAGE protein marker 10-250kD from Epizyme, Shanghai, China.

LB medium (BD, Shanghai, China).

Crystal screen kit: Crystal Screen 1/2 from Hampton, US; PEGs and PACT from NeXtal, US.

4-20% precast gel (Tanon, Shanghai, China), 20× MOPS Running buffer (Tanon, Shanghai, China).

5× SDS PAGE sample loading buffer is self-prepared as 250 mM Tris-HCl (pH6.8), 10% (w/v) SDS, 0.5% (w/v) bromophenol blue, 50% (w/v) glycerol and 5% (w/v) 2-ME were resolved and diluted by ddH_2O and then stored at -20°C.

Supplementary Data

Table S1 sequence

protein	sequence
mCherry	GMVSKGEEDNMAIIKEFMRFKVHMEGSVNGHEFEIEGEGEGRPYEGT<br>QTAKLKVTKGGPLPFAWDILSPQFMYGSKAYVKHPADIPDYLKLSFP<br>EGFKWERVMNFEDGGVVTVTQDSSLQDGEFIYKVKLRGTNFPSDGPV<br>MQKKTMGWEASSERMYPEDGALKGEIKQRLKLKDGGHYDAEVKTTYK<br>AKKPVQLPGAYNVNIKLDITSHNEDYTIVEQYERAEGRHSTGGMDEL<br>YK
LaM1	GSAQVQLVESGGGLVQAGDSLRLSCAASGRTFENYAMGWFRQAPGKE<br>REFVGAVSWGGGRTYYADNVKGRFTISRDNAKKNTVYLQMNSLKPED<br>TAVYYCAAKSVLTIATMRVPDEYNYWGQGTQVTVS
LaM3	GSAQVQLVQSGGGLVQAGGSLRLSCAASGRTFSDIAVGWFRQTPGKE<br>REFVAAISWSGLIINYGDSVEDRFTISRDNAKSAVYLQMNSLKPEDT<br>AVYYCAARIGMNYYYAREIEYPYWGQGTQVTVSKCY
LaM8	GSAQVQLVESGGGLVQAGGSLRLSCAVSGRPFSEYNLGWFRQAPGKE<br>REFVARIRSSGTTVYTDSVKGRFSASRDNAKNMGYLQLNSLEPEDTA<br>VYYCAMSRVDTDSPAFYDYWGQGTQVTVSTPRS
LaM1-(GGGGS)$_4$-LaM8	QVQLVESGGGLVQAGDSLRLSCAASGRTFENYAMGWFRQAPGKEREF<br>VGAVSWGGGRTYYADNVKGRFTISRDNAKKNTVYLQMNSLKPEDTAV<br>YYCAAKSVLTIATMRVPDEYNYWGQGTQVTVSSGGGGSGGGGSGGGG<br>SGGGGSQVQLVESGGGLVQAGGSLRLSCAVSGRPFSEYNLGWFRQAP<br>GKEREFVARIRSSGTTVYTDSVKGRFSASRDNAKNMGYLQLNSLEPE<br>DTAVYYCAMSRVDTDSPAFYDYWGQGTQVTVSS
LaM8-(GGGGS)$_4$-LaM4	QVQLVESGGGLVQAGGSLRLSCAVSGRPFSEYNLGWFRQAPGKEREF<br>VARIRSSGTTVYTDSVKGRFSASRDNAKNMGYLQLNSLEPEDTAVYY<br>CAMSRVDTDSPAFYDYWGQGTQVTVSSGGGGSGGGGSGGGGSGGGGS

	QVQLVESGGSLVQPGGSLRLSCAASGRFAESSSMGWFRQAPGKEREF<br>VAAISWSGGATNYADSAKGRFTLSRDNTKNTVYLQMNSLKPDDTAVY<br>YCAANLGNYISSNQRLYGYWGQGTQVTVSS
His6-<br>SUMO-<br>DsRed	MGSSHHHHHHSSGLVPRGSHMASMSDSEVNQEAKPEVKPEVKPETHI<br>NLKVSDGSSEIFFKIKKTTPLRRLMEAFAKRQGKEMDSLRFLYDGIR<br>IQADQTPEDLDMEDNDIIEAHREQIGGSRSSKNVIKEFMRFKVRMEG<br>TVNGHEFEIEGEGEGRPYEGHNTVKLKVTKGGPLPFAWDILSPQFQY<br>GSKVYVKHPADIPDYKKLSFPEGFKWERVMNFEDGGVVTVTQDSSLQ<br>DGCFIYKVKFIGVNFPSDGPVMQKKTMGWEASTERLYPRDGVLKGEI<br>HKALKLKDGGHYLVEFKSIYMAKKPVQLPGYYYVDSKLDITSHNEDY<br>TIVEQYERTEGRHHLFL

The aligned sequence of LaM nanobodies, mCherry and DsRed

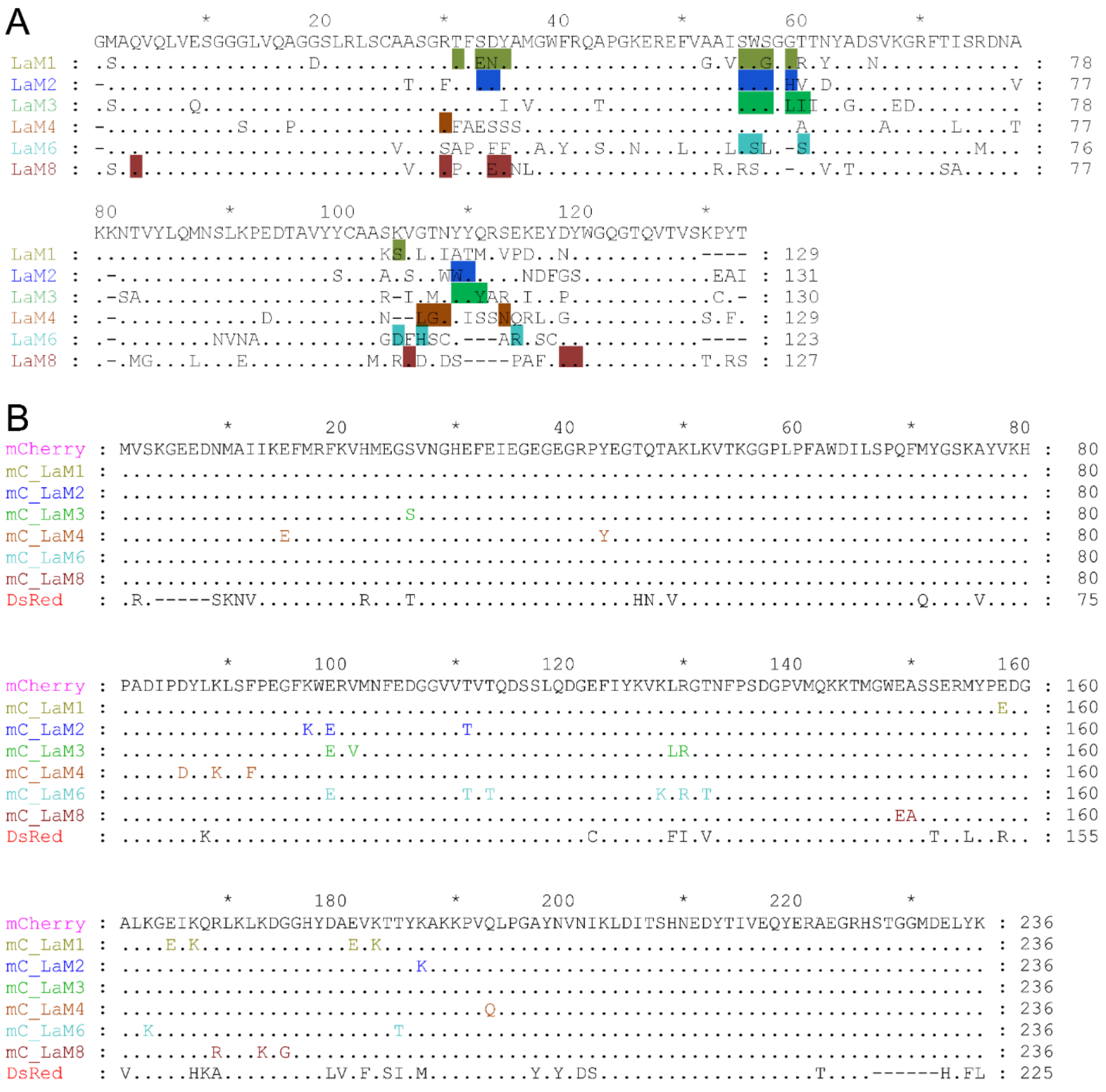

Fig. S1 Aligned sequence of LaM nanobodies, mCherry and DsRed. (A) The aligned sequence of LaMs. ClustalW aligned sequence of LaMs in MEGA-X, plotted by GeneDoc. A consensus sequence among LaMs was shown in the above alignment, the different residues were shown in upper letters. In contrast, the consensus residues were shown in dots, and the key residues of LaM binding to mCherry were highlighted. The critical residues of LaM2, LaM4, and LaM6 were from interface analysis of the PISA of PDBe. (B) The aligned sequence of mCherry and DsRed. The sequence of mC_LaM1,2,3,4,6,8 is mCherry (fasta data from Uniprot ID: X5DSL3), in which the residues of mCherry that binds corresponding LaM are shown as the upper letter. The residues of DsRed (fasta data from Uniprot ID: Q9U6Y8) different from mCherry are shown in upper letters. Other residues are shown as dots.

SDS-PAGE

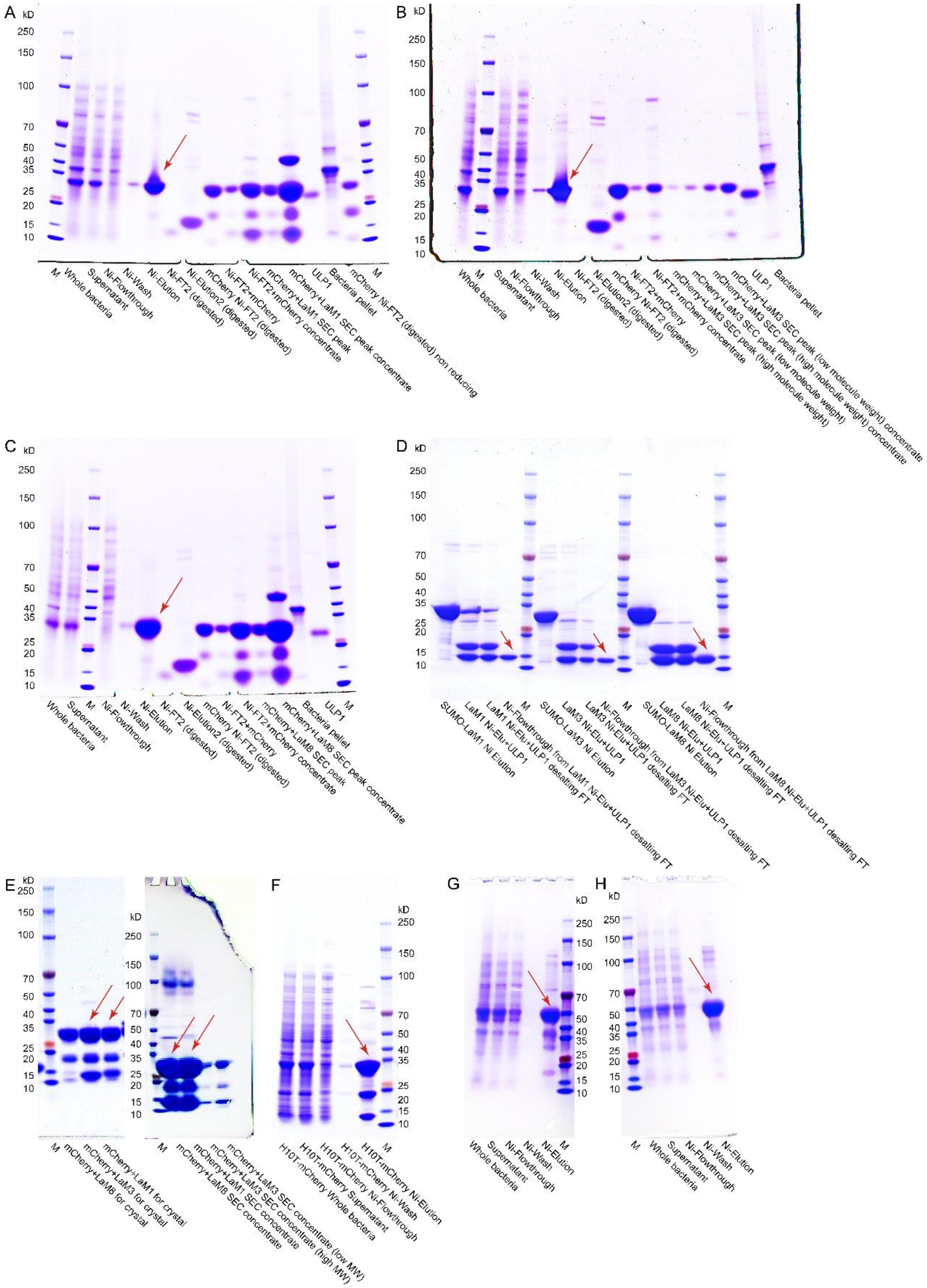

Fig. S2 SDS-PAGE for LaM nanobodies and mCherry. (A) SDS-PAGE for LaM1; (B) SDS-PAGE for LaM3; (C) SDS-PAGE for LaM8; (D) SDS-PAGE for LaM1, LaM3, LaM8 cleaved by ULP1， desalting, and Ni-flowthrough; (E) SDS-PAGE for complex

of protein for crystallization; (F) SDS-PAGE for H10T-mCherry; (G) SDS-PAGE for His-SUMO-LaM1-LaM8; (H) SDS-PAGE for His-SUMO-LaM8-LaM4.

Mass Spectrometry

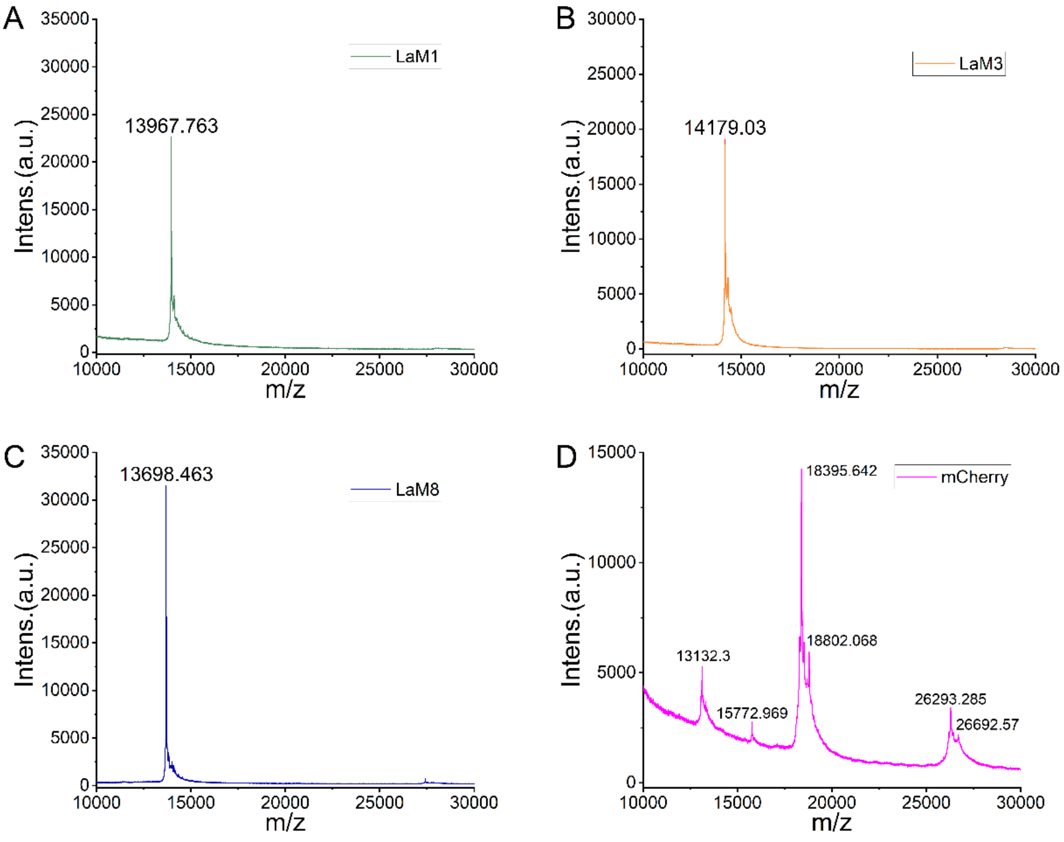

Fig. S3. Verify of LaM nanobody and mCherry by MALDI-TOF mass spectrometry. The x-axis represents the mass-to-charge ratio while the y-axis represents the detected intensity of the corresponding fragment in the sample. (A) LaM1; (B) LaM3; (C) LaM8; (D) mCherry.

Size Exclusion Chromatography

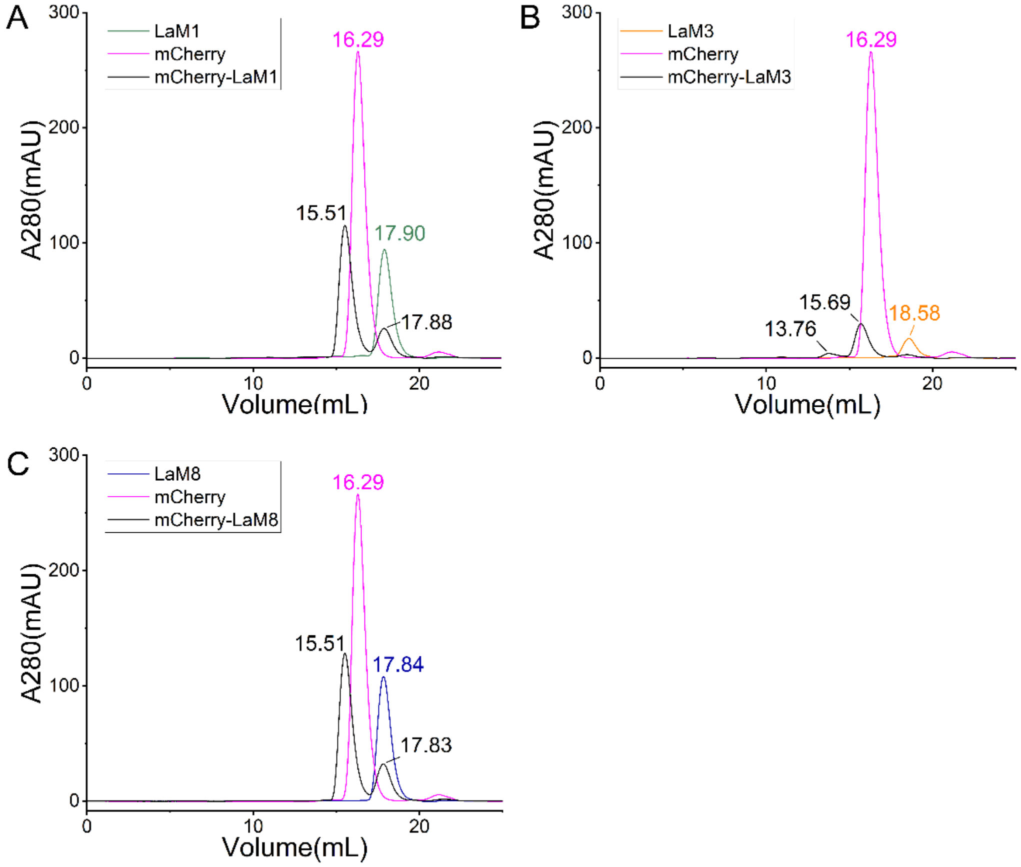

Fig. S4. Size exclusion chromatography of mCherry mix with LaM nanobodies. The x-axis represents the elution volume, while the y-axis represents the absorbance at 280nm. (A) The comparison of mCherry and LaM1 with their complex; (B) The comparison of mCherry and LaM3 with their complex; (C) The comparison of mCherry and LaM8 with their complex.

Bio-Layer Interferometry

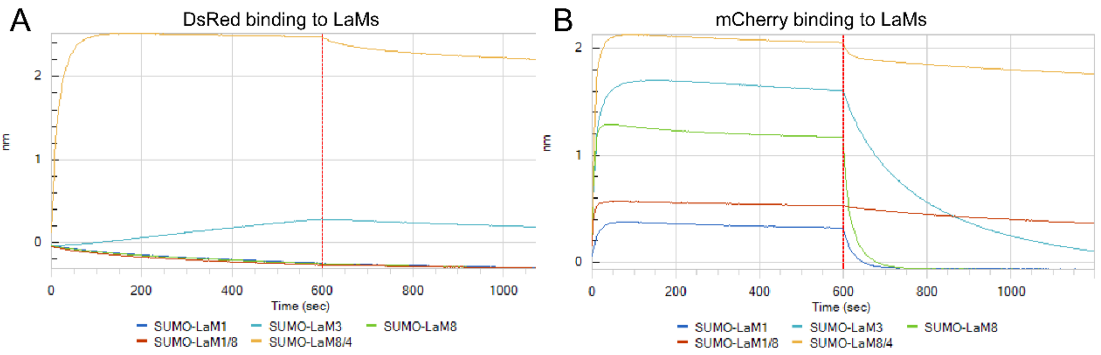

Fig. S5. The binding of mCherry or DsRed with LaM nanobodies by BLI. 1μM His-Sumo tagged proteins were loaded onto Ni-NTA biosensors, then equilibrated

before setting the baseline to zero at t=0. The association within 1μM DsRed (A) or 1μM mCherry (B) is in 0-600s, and the dissociation in kinetics buffer is in 600-1200s. The x axis represents time, and the y axis represents the thickness of protein binding to the biosensor surface.
